# Promoting and supporting leadership in hematology departments

**DOI:** 10.1002/hem3.70028

**Published:** 2024-10-17

**Authors:** Roch Houot, Emmanuel Gyan

**Affiliations:** ^1^ Department of Hematology University Hospital of Rennes Rennes France; ^2^ UMR U1236, INSERM University of Rennes, French Blood Establishment Rennes France; ^3^ Hematology and Cell Therapy Department University Hospital Tours France; ^4^ Clinical Investigation Centre, INSERM U1415 University Hospital Tours France

In recent years, the hospital system has faced tremendous pressure from economic and societal crises, which were further aggravated by the COVID‐19 pandemic. Today, the European healthcare system is in a continuous crisis, primarily due to underinvestment and workforce shortages.^1^ In such a situation, management skills at all levels of hospital departments become critical, especially for heads of departments—a position historically driven in many countries, including France, by academic rather than managerial competencies.

Challenges with medical staff retention have also exacerbated the workload of healthcare professionals.^2^ The shortage of healthcare workers in Europe is projected to reach 4.1 million by 2030, including 0.6 million physicians.^3^ Workforce shortages contribute to burnout among physicians and other healthcare workers, which renders the tasks of heads of departments extremely challenging.^4^, ^5^

In the past two decades, the practice of hematology has experienced accelerated advancements in diagnostics and therapeutics, with notable prolongation of patient survival, albeit at the cost of intensified medical care due to the novel time‐consuming therapeutic approaches. As such, the diversity of hematologic diagnoses and specialized treatments have created an expanding curriculum with ever more limited human resources.^2^, ^6^ The number of hematologic specialists and the competence of their training have become a concern in European countries.^6^

The European Hematology Association (EHA) has created solutions for training in hematology, including the European Hematology curriculum, developed through a “bottom‐up” process, which has inspired national educational initiatives.^6^ The European Working Time Directive (EWTD), introduced in 2004, aims to reduce long working hours to enhance patient safety. In this context, the European Commission urged member states to adopt the EWTD for hospital physicians.^2^ However, these initiatives create challenges for department heads, who must manage increased workloads with limited staffing and heightened awareness of the adverse effects of inadequate organization on workers' health.

In a recent survey conducted with 2390 university hospital faculty members in France between October and December 2021, 40% of participants had severe burnout, 14% had suicidal ideation, and 12% had job strain.^4^ The factors associated with the unfavorable experiences included heavy work overload, work‐life imbalance, and perceived lack of support from the institution.^4^, ^7^ Although the impact of stressful events on the risk of burnout and suicide is undeniable, many personality traits, such as emotional stability, extraversion, and social integration, play a role.^4^

In France, a group of heads of hematology departments from both academic and community hospitals gathered with the intuition that welcoming a community of heads of departments could provide support on an individual basis.^8^, ^9^ The community sets two main objectives: (i) an individual objective to support each head of department to achieve his/her mission; (ii) a collective goal to transform the hospital system starting from the “bottom‐up,” namely from the hospital departments. We will discuss the benefits of building this community of heads of hematology departments and some proposed solutions based on the evolving French experience, in the hope that this may be valuable for other medical departments and other European clinical hematology structures. A department, in this context, is a unit specialiszed in clinical hematology, sometimes within a larger division of internal medicine or medical oncology specialities.

## A NEW COMMUNITY OF THE HEADS OF HEMATOLOGY DEPARTMENTS

The initiative of creating a community of heads of hematology departments emerged in response to the French hospital system crisis and the challenges faced by the heads of departments. The community identified three directions: (i) reflecting on the missions of heads of departments and creating a network for them; (ii) sharing experience based on collective intelligence and brainstorming ideas to find solutions for the difficulties that each faces; and (iii) inventing and testing new forms of organization and management.^8^, ^9^ In French public hospitals, management training is sometimes offered but not mandatory for heads of departments, and most of them feel isolated from their counterparts.

## ACHIEVEMENTS OF THE EMERGING COMMUNITY

A 2‐day inaugural seminar was held in January 2023 in order to re‐evaluate the situation of hematology departments amidst the French healthcare crisis. This seminar established the foundational “core” group of 14 heads of departments. Throughout the year 2023, this emerging community gathered virtually on a monthly basis and launched a social network, offering sustained support to peers and adherence to the mission of the community, by sharing information and ideas.^8^

**Figure 1:**
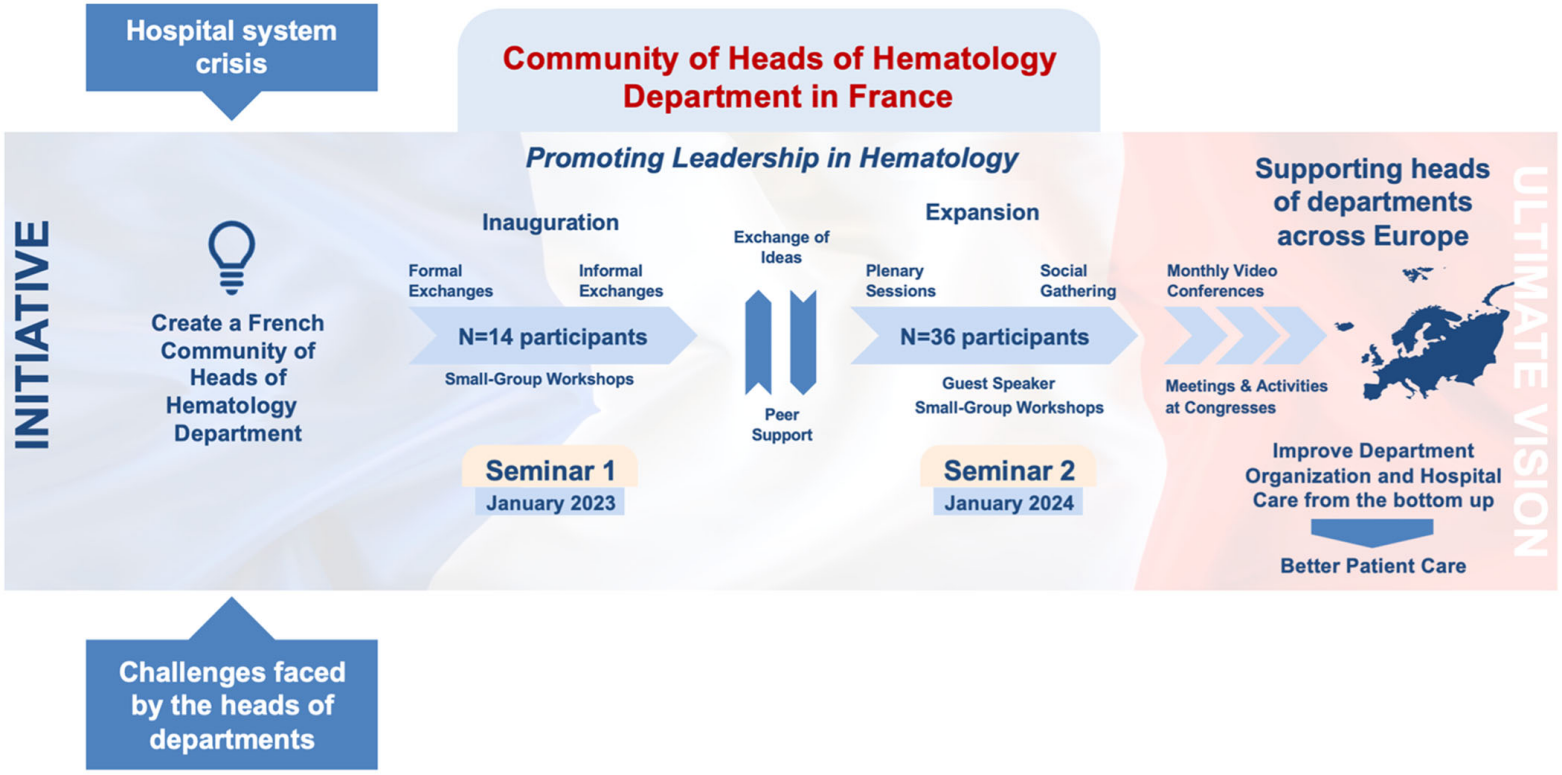
**The initiative of the community of heads of hematology departments in France**.

A second “expanded” seminar, held in January 2024, aimed to broaden the community by including additional heads of hematology departments.^9^ In total, 36 departments were represented, including university hospitals (69%), general hospitals (22%), and comprehensive cancer centers (9%). During that event, 36 heads of departments participated, exchanging their thoughts and reflecting on the mission of the community. Guest speakers were invited to share their experience, as well as external specialists in methods of co‐development and co‐construction in different settings (Figure 1).

The seminars were successful in promoting collective intelligence, creativity, strength, and support, highlighting the importance of regularly conducting these sessions to increase closeness and collaboration among heads of departments. These seminars expanded through continuous communication among the heads of departments via teleconferences, social network groups, working groups, and social gatherings.

## THEMES FREQUENTLY ENCOUNTERED DURING COMMUNITY MEETINGS

The inaugural seminar provided a platform for sharing management experiences among heads of departments, which, to our knowledge, is an unprecedented initiative at the specialist level in France. Different topics related to the organizational structure of the departments were tackled. The most noteworthy were: the methods adopted for involving medical and paramedical teams; cooperation and interaction between the head of department and staff managers (both paramedical and administrative); social interaction within the teams; induction and training of new employees; organization of departmental meetings; annual performance reviews; and sources of funding.^8^

In the second seminar, each participating head of a hematology department delivered a short presentation of 180 s to share an experience that could be useful for other heads of the department. The presentations addressed four main themes: management, patient care, research, and education/development. Participants were able to appreciate the challenges faced by their peers and the proposed solutions, as well as the learning opportunities from these experiences. Overall, the second seminar's presentations highlighted the importance of addressing the following topics in future sessions: exchanging and sharing problems and success stories; mutual aid and solidarity; reassurance and motivation; conviviality and meetings; and discovering management tools.^9^

## CURRENT CHALLENGES IN THE HOSPITAL SETTING IN FRANCE

Social support can provide encouragement, feedback, and positive reinforcement, which improves personal and professional relationships, and possibly increases stress tolerance. Indeed, a systematic review of 15 randomized clinical trials and 37 cohort studies evaluating the approaches to prevent and reduce burnout showed that social gatherings and facilitated small‐group programs decreased the prevalence of burnout among physicians from 54% to 44% (*p* < 0.001).^10^ Monthly dinner meetings improved engagement, human connection in the workplace, and the perceived sense of care by the department and supportive environment.^10^ This spontaneous bottom‐up approach may facilitate the emergence of robust solutions to problems widely faced by heads of departments from the same specialty. Indeed, having a group of heads of departments of the same specialty offers several advantages: (i) dealing with the same challenges specific to the specialty; (ii) having more opportunities to meet at scientific conferences and meetings related to the specialty; and (iii) the possibility of extending these exchanges through collaborative research initiatives.

Hospital organizations have encountered several challenges due to management practices that have not fully addressed functional and structural needs. For instance, in France, appointing medical heads of departments is predominantly based on academic credentials rather than managerial training and expertise. Heads of departments are also constrained by a highly hierarchical administrative system, resulting in limited freedom to manage their teams. Limited experience or understanding of administrative responsibilities, such as budgeting, resource allocation, staff management, or strategic planning, could be an additional source of constraints related to operational efficiency and financial management. Subsequently, inadequate managerial practices may negatively impact the professional's well‐being, the quality of patient care, and the effectiveness of research and education.

Given that heads of departments must lead interdisciplinary teams under budgetary constraints and practice bottom‐up governance, it is crucial to offer and ensure acceptance of leadership training and professional development opportunities for those with an academic focus. The inclusion of heads of departments in the decision‐making process is essential for organizational morale and performance, as they are at the forefront of hospital activity. Indeed, this approach contrasts with the current administrative organization of the hospital. The goal of the French community of heads of hematology departments is to enhance managerial skills, aiming to rebuild the healthcare system from the ground up. This initiative is in line with the French government's decision to create more autonomy in the medical departments, whereby the head of the department would be at the center of the hospital's management.^11^ This, in turn, implies that managerially trained heads of departments are prepared to participate in the multistakeholder organization that a hospital requires, in addition to their clinical and academic responsibilities. The head of department contributes massively to the internal structure of the department and the quality of life at work of the medical team, and constitutes the reference level in terms of organiszation, relevance, quality and safety of care, local management of medical and paramedical teams, and management of fellows and healthcare students.^12^ Our experience has demonstrated that a peer community is a powerful management support tool. It offers to its members the benefit from the collective intelligence, experience, creativity, strengths, and support of the group.

## CURRENT CHALLENGES IN THE LANDSCAPE OF HEMATOLOGY DEPARTMENTS

Hematology departments are experiencing staff shortages and an expanding curriculum, with the integration of promising new diagnostic and therapeutic options.^2^ For instance, the success of targeted therapies and immunotherapies has revolutionised the treatment landscape for various hematological disorders and improved patient outcomes, including prolonged survival. Hematologists consequently face new challenges that require ongoing research, multidisciplinary collaboration, and specialized expertise to optimize patient outcomes and ensure the safe and effective use of these therapies in clinical practice. Considering the complexities of the novel treatments and the increased number of cancer survivors, physicians are experiencing increased professional burdens. Serious consequences include burnout, which may ultimately compromise patient care.^13^ As talented physicians leave public hospitals to join alternatives that offer a better financial remuneration for better conditions, hematology departments are also suffering from staff shortages.^2^

Initiatives to increase investment in healthcare systems or hospital budgets to retain and attract healthcare professionals are necessary to counteract the effects of increased clinical overload. Another initiative includes the delegation of time‐consuming tasks to nonmedical personnel, including biomedical scientists in the laboratory and clinical nurse specialists, as observed in the National Health Service of the United Kingdom.^2^ There is increasing acceptance and encouragement of the latter in France^14^; however, the medical profession needs to be an active participant in this evolution. Interventions by professional societies and communities have an impactful role in preventing and managing increased burden on an individual level. Published research has highlighted the prevalence of burnout among European oncologists, and increased awareness campaigns in national and international congresses,^13^ and the EHA is addressing this issue amongst young hematologists.^15^ Data on burnout prevalence among European hematologists of all ages must be collected to call for investment in measures that make hematology an attractive field for both young and senior physicians.

## CONCLUSIONS AND FUTURE PERSPECTIVE

Healthcare authorities and hospital administrators should play a vital role in developing strategies to alleviate physician burnout in France and other European countries. However, this is far from reality. To that end, a bottom‐up rebuild of the hospital system is necessary to accommodate administrative changes. Engaging in supportive networks, such as the community we are establishing, allows for shared experiences and learning opportunities. This community of heads of hematology departments serves as a powerful tool for management support. Given the individual and organizational initiatives of this kind of community, heads of other specialty departments are encouraged to actively engage in community building and contribute to the reconstruction of hospital organization, structure, and functionality from the bottom up. Likewise, such engagement with heads of departments from other European countries will certainly contribute to sharing different care and organization models that inspire each other.

## AUTHOR CONTRIBUTIONS

The authors contributed to the writing and the editing of the article and approved its final version for submission and publication.

## CONFLICT OF INTEREST STATEMENT

The authors declare no conflicts of interest.

## FUNDING

The medical writing was funded by Kephren Publishing.

## Data Availability

Data sharing is not applicable to this article, as no new data were created or analyzed.
